# Biomass and Astaxanthin Productivities of *Haematococcus pluvialis* in an Angled Twin-Layer Porous Substrate Photobioreactor: Effect of Inoculum Density and Storage Time

**DOI:** 10.3390/biology8030068

**Published:** 2019-09-18

**Authors:** Thanh-Tri Do, Binh-Nguyen Ong, Minh-Ly Nguyen Tran, Doan Nguyen, Michael Melkonian, Hoang-Dung Tran

**Affiliations:** 1 Faculty of Biology, Ho Chi Minh City University of Education, 280 An Duong Vuong Street, District 5, Ho Chi Minh City 72711, Vietnam; 2 Faculty of Biotechnology, Nguyen-Tat-Thanh University, 298A-300A Nguyen-Tat-Thanh Street, District 04, Hochiminh City 72820, Vietnam; 3 Vietnam-United States-Australia Biotechnology Company, Group 4, Cay Da Ward, Nguyen Thi Lang Street, Cu Chi District, Hochiminh City 71609, Vietnam; 4 Faculty of Business and Law, Swinburne University of Technology, Mail H25 PO Box 218, Hawthorn, Victoria 3122, Australia; 5 Biozentrum Köln, Universität zu Köln, Zülpicher Str. 47 b, 50674 Köln, Germany

**Keywords:** *Haematococcus pluvialis*, astaxanthin, porous substrate photobioreactor, a horizontal (low-angled) twin-layer photobioreactor

## Abstract

The microalga *Haematococcus pluvialis* is mainly cultivated in suspended systems for astaxanthin production. Immobilized cultivation on a Twin-Layer porous substrate photobioreactor (TL-PSBR) has recently shown promise as an alternative approach. In Vietnam, a TL-PSBR was constructed as a low-angle (15 °) horizontal system to study the cultivation of *H. pluvialis* for astaxanthin production. In this study, the biomass and astaxanthin productivities and astaxanthin content in the dry biomass were determined using different initial biomass (inoculum) densities (from 2.5 to 10 g dry weight m^−2^), different storage times of the initial biomass at 4 °C (24, 72, 120 and 168 h) and different light intensities (300–1000 µmol photons m^−2^ s^−1^). The optimal initial biomass density at light intensities between 400–600 µmol photons^−2^ s^−1^ was 5–7.5 g m^−2^. Algae stored for 24 h after harvest from suspension for immobilization on the TL-PSBR yielded the highest biomass and astaxanthin productivities, 8.7 g m^−2^ d^−1^ and 170 mg m^−2^ d^−1^, respectively; longer storage periods decreased productivity. Biomass and astaxanthin productivities were largely independent of light intensity between 300–1000 µmol photons m^−2^ s^−1^ but the efficiency of light use per mole photons was highest between 300–500 µmol photons m^−2^ s^−1^. The astaxanthin content in the dry biomass varied between 2–3% (w/w). Efficient supply of CO_2_ to the culture medium remains a task for future improvements of angled TL-PSBRs.

## 1. Introduction

Recent studies have shown strong antioxidant activity of astaxanthin [1] with benefits to the immune system, cardiac muscles, and to treatments of cancer and skin aging [1,2,3,4]. The green alga *Haematococcus pluvialis* is the most widely used source for natural astaxanthin at a commercial scale and has been reported to accumulate astaxanthin up to 5.9% (w/w) of the dry biomass [5,6,7].

Due to its life history, *H. pluvialis* is usually grown in two-phase cultivation, in which the cells are grown by cell multiplication in the “green phase” and are stimulated to accumulate astaxanthin in the successive “red phase” (e.g., [6,8]). The two-phase cultivation method is technically demanding and is characterized by high energy consumption and relatively long cultivation times [9,10].

Two-phase suspended cultivation of *H. pluvialis* is the predominant cultivation system at both exploratory and commercial scales. In suspended cultivation, a maximum light intensity of 150 µmol photons m^−2^ s^−1^ should not be exceeded to maintain cell growth and divisions and environmental parameters such as temperature, CO_2_, and pH need to be closely monitored [6,8]. As the needed biomass is reached, the second phase, known as the stressed or red phase is switched on to stimulate astaxanthin accumulation [6,8]. Suspended cultivation of *Haematococcus* is performed in open ponds/raceways or in closed tubular or flat-plate photobioreactors (PBR). Open pond cultivation is usually used only for the stressed phase with short cultivation time (4–6 days) to minimize contamination and apply stress conditions [8]. The closed PBR can minimize contamination and control growth parameters better, but has the drawbacks of high assembly and maintenance costs (on average, a tenfold increase in costs) [11,12,13]. Suspended cultivation usually yields low biomass density (0.05–0.06% of the total cultivation liquid) due to the ineffective light and carbon dioxide distribution caused by the low surface-to-volume ratio of the cultivation systems and demands additional effort for the harvest of the algae [14]. Flat or tubular closed systems increase productivity by increasing the efficiency of light usage and limiting contamination, but there are other limitations, especially in the adhesion of cells to the inner PBR surfaces of many microalgae including *H. pluvialis* [15].

Porous substrate photobioreactors (PSBR) such as the vertical Twin-Layer system can be used to cultivate various algal species including *H. pluvialis* (for a review, see Podola et al. [16]). PSBRs are a recently developed technique for microalgal cultivation in immobilized systems that has also shown considerable promise for application in *Haematococcus* [7,17,18,19,20,21]. The Twin-Layer system is a modular PSBR. The prototype (large scale) TL-PSBR is capable to operate up to eight Twin-Layer modules, the area for growth of microalgae on both sides is 2 × 0.67 m^2^ for each module. A tube-type TL-PSBR for small-scale holding of 12 Twin-Layer modules of 10 × 90 cm was used to monitor growth kinetics of *H. pluvialis* [15,22,23]. Each TL module consists of a vertically orientated source layer (e.g., glass fiber nonwoven), through which a flow of medium is established to feed nutrient. Onto both sides of the source layer, the substrate layers (e.g., printing paper or filter paper that has small porosity) are applied by self-adhesion of hydrogen bonds. Microalgae will be immobilized on the substrate layers and will develop a biofilm [15,22,23].

The concurrent production of biomass and astaxanthin of *H. pluvialis* in one-phase cultivation at high light intensity with CO_2_ supplementation using a vertical Twin-Layer photobioreactor yielded biomass productivities of up to 19.4 g m^−2^ d^−1^ and final biomass of 213 g dry weight m^−2^ growth area after 16 days of cultivation [23]. In comparison with two-phase cultivation using the same photobioreactor system, one-phase cultivation yielded a similar astaxanthin output in just half of the cultivation time [23]. In one-phase cultivation on TL-PSBR, growth and astaxanthin accumulation of *H. pluvialis* happens simultaneously but in different cell layers of the biofilm. Cells on the surface are exposed to direct light and change into red akinetes, whereas green cells in the lower layers divide by spore formation [23]. Hence, the thickness of the biofilm and high light intensity play an important role in one-phase cultivation. However, in modular vertical TL-PSBRs high intensity and uniformity of incident light is difficult to achieve due to mutual shading by adjacent modules, although the vertical arrangement of the TL modules saves space and enhances areal productivity. 

A horizontal (low-angled) TL-PSBR was developed for research purposes in Vietnam starting in 2016, as it is easier to construct and operate than a vertical TL system and for high light applications such as the production of astaxanthin, only slightly less productive (aerial productivity) than a vertical system. Furthermore, harvesting the biomass is technically less complicated in low-angled TL-systems compared to vertical systems [16]. In Vietnam, the angled Twin-Layer photobioreactor system for immobilized cultivation of microalgae provides significant advantages over traditional suspended cultivation such as saving of water, energy and cultivation time [24]. This study investigates several factors affecting biomass and astaxanthin accumulation of *H. pluvialis* cultivated in a small-scale angled TL-PSBR: initial biomass (inoculum) density, algae storage time before immobilization and influence of different light intensities. Furthermore, a larger-scale angled TL-PSBR system for *H. pluvialis* was designed and operated to probe upscaling astaxanthin production to pilot scale.

## 2. Materials and Methods

### 2.1. Algal Strain and Culture Maintenance 

The *H. pluvialis* strain CCAC 0125 was supplied from the Culture Collection of Algae at the University of Cologne, Germany (http://www.ccac.uni-koeln.de/). The strain was maintained in 100 mL Erlenmeyer flasks, with 50 mL of modified BG-11 medium [23] and transferred every 3–4 weeks. Cultures were grown at 20 °C, with a light intensity of 30 µmol photons m^−2^ s^−1^ provided by fluorescent lamps (Philips Essential energy saver 18W E27 Cool day light lamp, Philips Lighting GmbH, Hamburg, Germany), using a 14/10 h light/dark cycle.

### 2.2. Experimental Designs

The experiments were carried out on small scale (0.1 × 0.5 = 0.05 m^2^ growth area) and large scale (0.5 m^2^ × 4 growth area) angled TL-PSBRs [24]. The non-sterile culture medium was stored in 40 L plastic chambers.

#### 2.2.1. Experiments with the Small-Scale TL-PSBR

**Figure 1:**
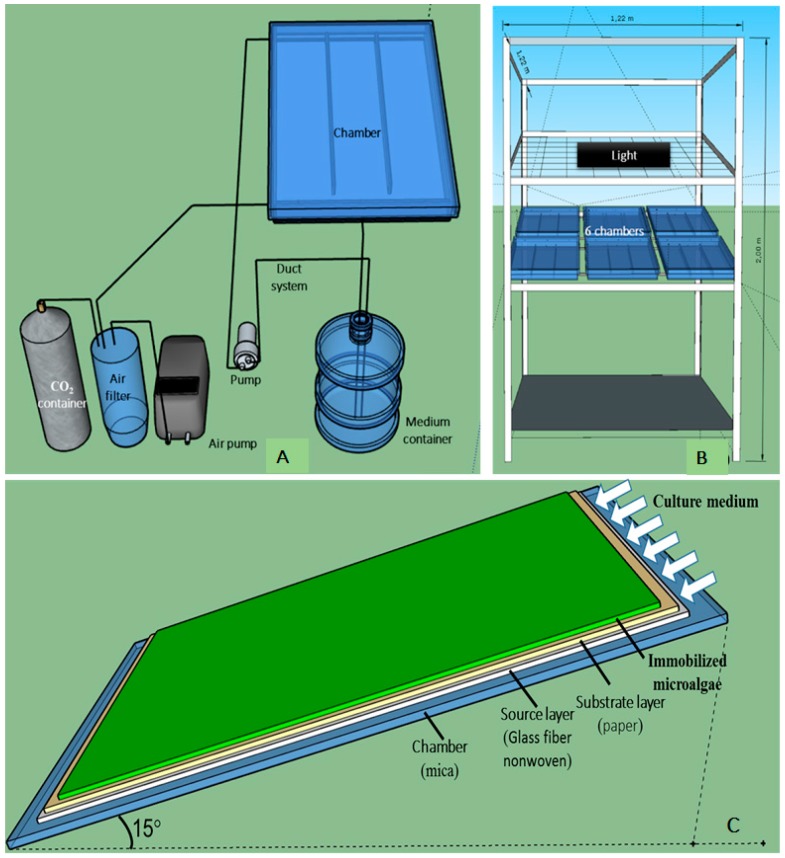
The small-scale angled TL-PSBR: nutrient and air supply system for cultivation chamber (**A**), positioning of chambers and lights (**B**), components inside a chamber (**C**).

The small-scale angled TL-PSBR for *H. pluvialis* immobilized cultivation includes small chambers with source layers, substrate layer (paper) and immobilized algae inside, nutrient circulation system, air circulation system, light supply system and steel frame (Figure 1).

Each chamber is 0.37 × 0.52 m in size divided into three compartments. Each compartment is about 0.12 × 0.52 m in size and contains three layers: the source layer (nonwoven fiberglass, 0.11 × 0.51 m), substrate layers (Kraft paper 70 g m^−2^, 0.11 × 0.51 m) and biofilm (immobilized algae on the substrate layer, the growth area, 0.1 × 0.5 m) (Figure 1C). A small-scale TL-PSBR has six small chambers (Figure 1B) that share the same nutrient, air and light supply systems. Therefore, a small system with 18 growth areas (0.05 m^2^ for each) for microalgae was divided into different treatments for each experiment.

Three experiments with the small-scale TL-PSBR were carried out. The most effective outcome from each preceding experiment would be applied for the next experiment.

Influence of algal storage time before immobilization: the concentrated algae were stored at 4 °C in the dark over 24, 72, 120 and 168 h after centrifugation, respectively. Initial algae biomass for inoculation of the TL-PSBR was 5 g dry weight m^−2^, the light intensity was adjusted to reach the range of 400–600 µmol photons m^−2^ s^−1^ over the growth area.

The optimal initial algal density: the initial biomass densities of 2.5, 5, 7.5 and 10 g dry weight m^−2^ were tested. Algae were immobilized immediately after centrifugation or were stored for 24 h at 4 °C. The light intensity was set at 400–600 µmol photons m^−2^ s^–1^.

Influence of light intensity on algal growth and astaxanthin accumulation: The experiment used sodium high-pressure lamps (PHILIPS) as a light source with the intensity of 300–1000 µmol photon m^−2^ s^−1^. Each point on the cultivation chambers received different light intensity and was considered to be one experiment for one value point of this parameter. The initial biomass density was 7.5 g dry weight m^−2^.

#### 2.2.2. Experiment with the Large Scale TL-PSBR

**Figure 2:**
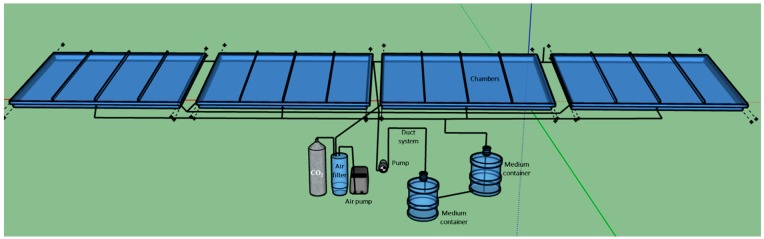
Design of the nutrient and air supply system for the large-scale angled TL-PSBR.

The large-scale angled TL-PSBR uses the same components of the small system but has four larger chambers assembled in the same nutrient, air and light supply systems, each chamber provides 1 × 0.5 = 0.5 m^2^ area for algae growth (Figure 2). Thus, each large-scale TL-PSBR will have 0.5 × 4 = 2 m^2^ growth area. Two large TL-PSBRs with two different lighting systems were set up and operated at the same time to perform the experiment.

In large-scale TL-PSBRs, the experiment was set up to compare the efficiency for lighting of two different luminaires. The two luminaires used to compare energy efficiency in this experiment are: luminair 250W (light intensity of 300–1150 µM photons m^−2^ s^−1^, provided by ten PHILIPS 250W sodium high-pressure lamps) and luminair 400W (light intensity of 300–1300 µmol photon m^−2^ s^−1^, provided by eight PHILIPS 400W sodium high-pressure lamps).

Some experimental conditions were established based on the results obtained with the small TL-PSBR, in which the initial dry biomass density was 7.5 g m^−2^, and algae were immobilized after centrifugation within 24 h.

### 2.3. Preparation of H. pluvialis Biomass for Experiments on Angled TL-PSBRs

**Figure 3:**
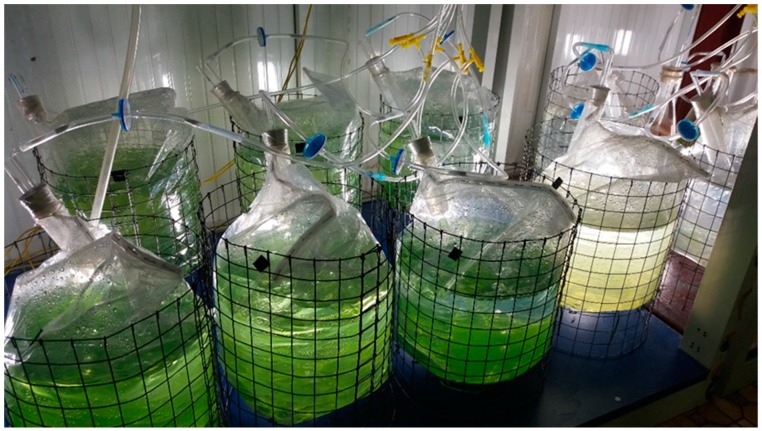
Suspended cultivation of *H. pluvialis* in 10 L PE bags.

Suspended cultivation before immobilization: The culture medium used was modified BG-11 [23]. Suspended cultivation of microalgae was done in 500 mL (culture time is about 10 days) and 2000 mL (culture time is about 14 days) Erlenmeyer flasks at 23 ± 2 °C, illuminated by a fluorescent lamp at 40–60 µmol photons m^−2^ s^−1^, using a 14/10 h light/dark cycle. However, the large-scale angled TL-PSBRs required a larger amount of initial algal biomass, so the volume of suspended algae was increased to 10 L in PE bags (with 6 L of modified BG-11 medium) (Figure 3). Algae from 2000 mL flasks will be transplanted through PE bags with the initial algal density of about 1.5 x 10^4^ cells mL^−1^, and a culture period of about 16 days. The medium was aerated with ambient air during the cultivation period, illuminated by fluorescent lamps at 40–60 µmol photons m^−2^ s^−1^ with 14/10 h light/dark cycle, at 23 ± 2 °C.

Centrifugation to concentrate algae in suspension: Algae in 10 L PE bags after 20 days of culture consisted to 85% of flagellate cells and were harvested from suspension by low-speed centrifugation (800 × *g* for 5 min) with a large bench centrifuge (ROTANTA 460 RC, Hettich GmbHo. KG, Tuttlingen, Germany). Under these conditions, no damage was observed to the flagellate cells. After centrifugation, the concentrated algal suspension was used to determine the amount of dry biomass (mL). 

The dry biomass of the concentrated algal suspension was determined by filtering 1 mL of the suspension onto a filter paper (0.4 µm pore size, Whatman GmbH, Dassel, Germany) with a predetermined dry weight of W_1_ and dried the whole set in 2 h at 105 °C. The dried filter paper with algae was weighed, and the drying process repeated until an unchanged total weight of W_2_ was achieved. The dry biomass in 1 mL concentrated algae liquid was calculated as D = W_2_ – W_1_.

### 2.4. Algae Immobilization on Angled TL-PSBRs

The inoculation of algae on the substrate layer was tested with several methods. However, using a brush to apply the concentrated algal suspension x showed many advantages. The required volume V (mL) of algal suspension immobilized onto a surface area S was calculated as V (mL) = (M × S)/D with M as the initial immobilized dry biomass density (g m^−2^). The concentrated algal suspension was immobilized by a soft brush onto substrate layer on 0.05 and 0.5 m^2^ TL-PSBRs, respectively.

### 2.5. Culture Conditions after Algal Immobilization

Immobilized cultivation time was 10 days. A nutrient-replete BG-11 medium was used during days 1–7 by replacing 20 L (on small-scale TL-PSBR) or 40 L (on large scale TL-PSBR) of medium every 2 or 3 days. Twenty or forty litres of BG-11 medium without N and P (excludes NaNO_3_ and K_2_HPO_4_ × 3H_2_O in modified BG-11 medium) [18] were used on days 8, 9 and 10 for astaxanthin induction. The culture medium was supplemented with CO_2_ 1% (v/v) as a carbon source and also as a means for pH adjustment. The temperature was kept in 23–26 °C, pH = 6.5–8. The electrical conductivity of the medium was kept in 1800–2000 µS cm^−1^ by adding filtered water after every 24 h.

The following variables were monitored: Dry algae biomass productivity (g m^−2^ d^−1^) was calculated based on equation M_p_ = (M_a_-M)/10 (M_a_: dry biomass after 10 days, M: initial immobilized dry biomass); astaxanthin productivity (mg m^−2^ d^−1^) was calculated based on equation A_p_ = (M_a_ × 1000 × %A)/10, %A is the astaxathin content in the dry biomass.

Measurement of immobilized dry biomass: after 10 days of cultivation, the thin algal layer and substrate layer (Kraft paper 70 g/m^2^, Vietnam) were harvested and dried for 2 hours at 105 °C, and cooled in a desiccator in 30 min. The dried product was weighed and the drying process repeated until an unchanged total weight of m_2_ was obtained. The dry biomass was calculated as M_a_ = m_2_ − m_1_ (g), m_1_ is the weight of dry paper without algae.

Measurement of astaxanthin content in the dry biomass (%A): Astaxanthin was determined spectrophotometrically according to Li et al. [25]. All processes were carried out in the dark. A sample of 1 mg of freeze-dried biomass was placed with 0.5 mL acetone 90% in a 2 mL tube with screw cap to limit the vaporization, incubated in water bath at 70 °C for 5 min with intervals for vortexing, and then ground using a glass pestle. The mixture was centrifuged at 4000× *g* for 5 min and the supernatant retrieved. Extraction with acetone was repeated until the pellet turned white. The extract was supplied with acetone (90%) to reach a volume of 3 mL in a 5 mL sealed centrifuge tube with screw cap until spectrophotometric analysis.

**Figure 4:**
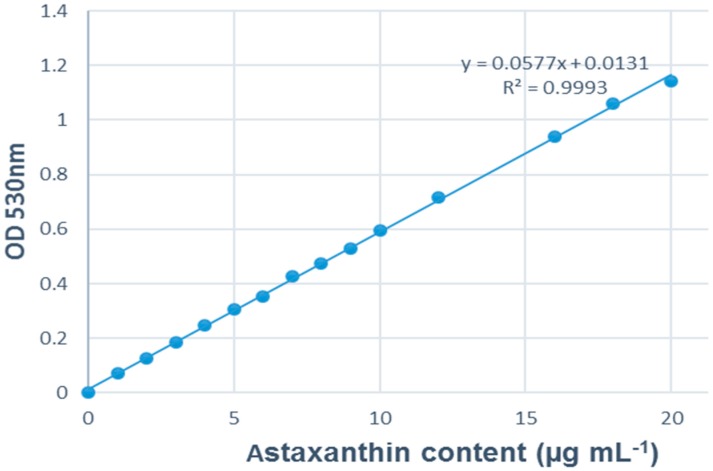
Astaxanthin standard calibration curve for spectrophotometry.

The OD value of the extracted pigments was measured at a wavelength of 530 nm with Ultrospec 2100 Pro Spectrophotometer (Amersham Biosciences). Astaxanthin content was calculated based on the calibration curve equation of y = 0.0577x + 0.0131 (established with astaxanthin ≥ 98 % (HPLC), Sigma-Aldrich, St. Louis, MO, USA) (Figure 4), in which y was the OD value measured and x was the astaxanthin content (µg mL^−1^). Astaxanthin content in biomass was calculated in percentage (A%), and subsequent estimation of astaxanthin production per biofilm surface (in mg m^−2^ d^−1^).

### 2.6. Data Analysis

The data were analyzed using Microsoft Excel 2016. Statistical analysis and resulting charts were made using R program version 3.4.2. The presented values were averaged value of more than three replicates with corresponding standard deviation.

## 3. Results

### 3.1. Dry Biomass Growth and Astaxanthin Accumulation of Immobilized H. pluvialis Cultivated in an Angled TL-Photobioreactor at Different Initial Biomass Density

**Figure 5:**
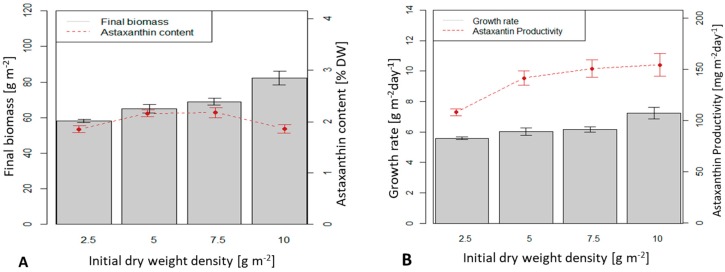
Final biomass and astaxanthin content in dry weight (%) in *H. pluvialis* at different initial biomass densities after 10 days of growth in a TL-BSBR. (**A**) Final biomass and astaxanthin content; (**B**) Biomass growth rate (average of biomass increase in 10 days) and astaxanthin productivity.

At an initial biomass density of 2.5, 5 and 7.5 g m^−2^, the biomass productivities were 5.6, 6 and 6.2 g m^−2^ d^−1^, respectively (Figure 5B). These values were not significantly different from each other. The highest average dry biomass productivity reached 7.23 g m^−2^ d^−1^ at an initial biomass density of 10 g m^−2^ and was significantly different from the other three values (*p* < 0.05).

Final astaxanthin content in the dry biomass reached the highest value of 2.17 % at initial biomass densities of 5 and 7.5 g m^−2^, respectively. However, the astaxanthin content at lower (2.5 g m^−2^) or higher (10 g m^−2^) initial biomass densities was lower (1.87 %) and the difference was statistically significant (*p* < 0.05).

Except for low initial biomass densities (2.5 g m^−2^), astaxanthin productivities at initial biomass densities of 5, 7.5 and 10 g m^−2^ were similar: 141 (5 g m^−2^), 151 and 154 mg.m^−2^ d^-−1^ (at 7.5 and 10 g m^−2^).

### 3.2. Influence of Algal Storage Time after Centrifugation of a Suspension Culture on Biomass Growth and Astaxanthin Accumulation in a TL-PSBR

After a storage time of 24 hours before immobilization and at an initial biomass density of 5 g m^−2^, the highest value of average dry biomass productivity after 10 days of cultivation was achieved (8.75 g m^−2^ d^−1^). At an initial biomass density of 5 g m^−2^, storage times of 72, 120 and 168 h yielded average productivity of 6.3, 1.7 and 2.7 g m^−2^ d^−1^, respectively. Biomass productivities at storage time of 24, 72 and 120 h were significantly different (*p* < 0.05) while productivities at storage times of 120 and 168 h were not (*p* > 0.05).

**Figure 6:**
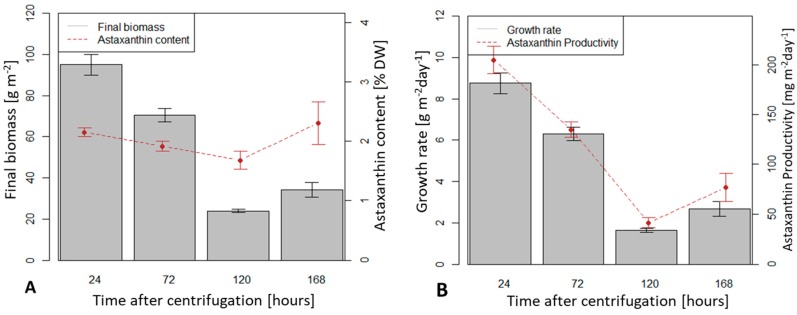
Biomass and astaxanthin in *H. pluvialis* at different storage times of initial biomass after centrifugation from suspension culture. (**A**) Final biomass and astaxanthin content; (**B**) Growth rate (average of biomass increase in 10 days) and astaxanthin productivity.

Average astaxanthin content reached 2.15 % dry biomass at storage time of 24 h and an initial density of 5 g.m^−2^. It decreased with increased time storage until 168 h when it was higher again (1.92, 1.68 and 2.30 % at 72, 120, 168 h storage time, respectively). However, there was no significant difference between these values. Storage time did not influence the final astaxanthin content (% in dry weight) but only the final biomass content (Figure 6A). Consequently, the storage time of 24 h yielded the highest astaxanthin productivity (205 mg m^−2^ d^−1^) measured, astaxanthin productivity decreased significantly at longer storage times (Figure 6B).

### 3.3. Influence of Light Intensity on Biomass Growth and Astaxanthin Accumulation of H. pluvialis in an Angled TL-PSBR

The effect of different light intensities was studied in the range of 300–1000 µmol photons m^−2^ s^−1^ in 8 experiments.

A light intensity of 400 µmol photons m^−2^ s^−1^ yielded the highest average dry biomass productivity (6.6 g m^−2^ d^−1^) and 300 µmol photons m^−2^ s^−1^ yielded the lowest productivity (5.56 g m^−2^ d^−1^). However, the productivity values in the whole range of 300–1000 µmol photons m^−2^ s^−1^ were not significantly different (*p* > 0.05).

**Figure 7:**
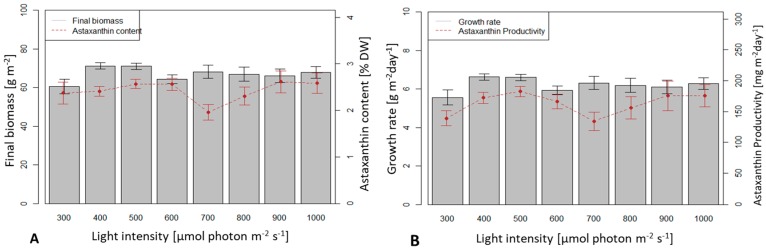
Biomass productivity and astaxanthin in *H. pluvialis* under different values of light intensities in the range of 300–1000 µmol photons m^−2^ s^−1^. (**A**) Final biomass and astaxanthin content; (**B**) Biomass growth rate and astaxanthin productivity.

Intensities of 500 and 600 µmol photons m^−2^ s^−1^ yielded highest astaxanthin content (2.56 %) and 700 µM photons m^−2^ s^−1^ yielded lowest value (1.96 %), the difference, however, was not statistically significant (*p* > 0.05) (Figure 7).

The highest average astaxanthin productivity reached 183 mg m^-2^ d^-1^ at 500 µmol photons m^−2^ s^−1^ but it was not significantly different from the value at other light intensities.

**Figure 8:**
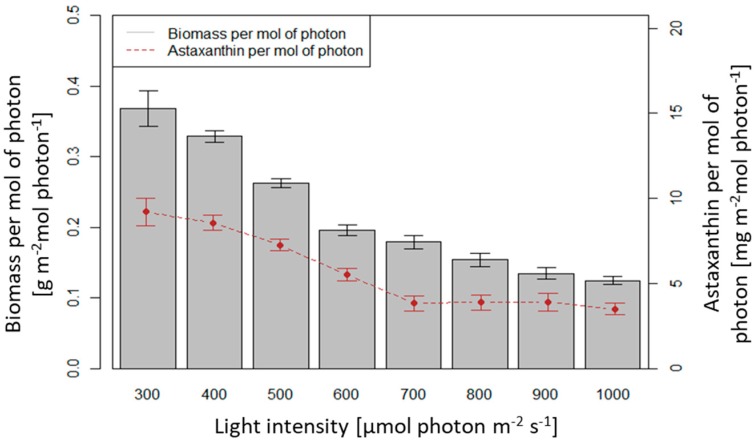
Dry biomass productivity and astaxanthin content in *H. pluvialis* per mole of photons at different values of light intensity in the range of 300–1000 µmol photons m^−2^ s^−1^.

In general, with a total of 140 h of illumination in 10 days, the dry biomass productivity per each mole of photons decreased with increased light intensity, therefore the highest light intensity only yielded the productivity of 0.12 g m^−2^ mol photons^−1^. The intensities of 300 and 400 µmol photons m^−2^ s^−1^ yielded 0.37 and 0.33 g m^−2^ mol photons^−1^, respectively (*p* > 0.05), which was significantly higher than at the other light intensities (*p* < 0.05). The light intensity of 500 µmol photons m^−2^ s^−1^ yielded 0.262 g m^−2^ mole photons^−1^, higher than the value of 600–1000 µmol photons m^−2^ s^−1^ (*p* < 0.05) (Figure 8).

Astaxanthin productivity per each mole of photons decreased with increased light intensity. At the intensities of 300, 400 and 500 µmol photons m^−2^ s^−1^ the productivities were 9.2, 8.5, 7.2 mg m^−2^ mole photons^−1^, respectively (*p* > 0.05) (Figure 8). These values were significantly higher than at other intensities (p < 0.05). The lowest astaxanthin productivity per mole of photons was 3.5 mg m^−2^ mole photons^−1^ at 1000 µmol photons m^−2^ s^−1^.

### 3.4. Influence of the Light Source on H. pluvialis Immobilized Cultivation in a 0.5 m^2^ x 4 (Large Scale) Twin-Layer Photobioreactor

**Figure 9:**
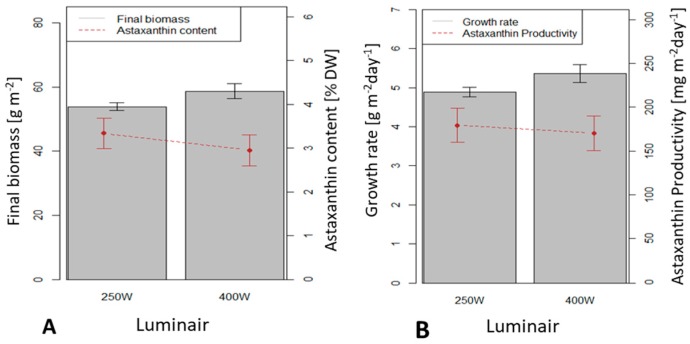
Dry biomass productivity and astaxanthin content and productivity in *H. pluvialis* with two different light sources. (**A**) Final biomass and astaxanthin content; (**B**) Biomass growth rate and astaxanthin productivity.

This study investigated the illumination efficiency of two different luminaires: luminair 250 W and luminair 400 W. The luminair 400 W yielded average dry biomass productivity of 5.4 g m^−2^ d^−1^, significantly higher than the productivity under luminair 250 W (4.8 g m^−2^ d^−1^) (*p* < 0.05). Astaxanthin contents in biomass under luminair 250 W and luminair 400 W (3.34 and 3.13 %, respectively) were not significantly different (*p* > 0.05) (Figure 9).

Astaxanthin productivities using luminair 250 W and 400 W (179.4 and 180.8 mg m^−2^ d^−1^, respectively) were not significantly different (*p* > 0.05). The luminair 400 W yielded higher dry biomass productivity but lower astaxanthin content than the other; therefore astaxanthin productivities were similar under both luminaires.

## 4. Discussion

At the lab scale, small-scale vertical TL systems were used to monitor growth kinetics and the influence of several factors such as initial density, light intensity, CO_2_ and nutritent concentrations on the growth and accumulation of astaxanthin of *H. pluvialis* in biofilms [18].

Here, we extended these studies to an angled TL system likely more suitable for applications in Vietnam.

### 4.1. Influence of Initial Biomass Density

Growth of *Haematococcus pluvialis* in a TL-PSBR under nutrient-replete conditions is characterized by two different processes: (1) growth by cell enlargement without cell divisions (in akinetes), and (2) growth by cell multiplication (spore formation in green cells). Both processes occur simultaneously (and over several days with similar biomass growth rates) but in different layers of the biofilm [23]. In the upper layers (that are exposed to high light intensities) red akinetes enlarge, whereas in the lower layers green cells divide by spore formation (in immobilized systems the spores are non-flagellate). In suspension culture, the organization of cell layers similar to immobilized culture was also used in a small-scale “double-layered” photobioreactor [10]. In that system, cells in the outer jacket region were exposed to excessive irradiation (770 µmol photons m^−2^ s^−1^) and accumulated astaxanthin in the biomass to reach 5.79 % of the dry weight. Meanwhile, with attenuated light energy (40 µmol photons m^−2^ s^−1^) and sufficient nutrients cells in the inner core region continued to grow by cell division to provide the inoculum for the next batch run [10].

An inoculum from a growing suspension culture usually consists of a majority of flagellate cells. After inoculation on a TL-PSBR these transform readily into the palmella stage. Almost 100% of the cells shed their flagella during cell immobilization and enhanced acclimation to high light was associated with this stage [26]. Cells on the top of the biofilm then accumulate astaxanthin and transform into red akinetes that protect cells in the lower layers. If the initial biomass (inoculum) density is very low (and the light intensity very high, i.e., >600 µmol photons m^−2^ s^−1^), reformation of green cells in the lower layers may be delayed for days until astaxanthin accumulation in the upper layers lowers the flux of blue photons to a level suitable for re-greening of akinetes [23,26]. It is, therefore, important to determine the minimum dry biomass (inoculum) density that allows the establishment of both red and green layers quickly after inoculation for a given light intensity. Research results on the vertical TL system determined the appropriate initial density for *H. pluvialis* to be 5 g m^−2^ and the final biomass of 213 g dry weight m^-2^ growth area after 16 days of cultivation. On angled TL-PSBRs in Vietnam, four initial microalgae densities (2.5, 5, 7.5 and 10 g m^−2^) were tested for selecting the appropriate initial density to increase biomass and astaxanthin of *H. pluvialis* simultaneously but with a 10-day culture period to limit infection.

**Figure 10:**
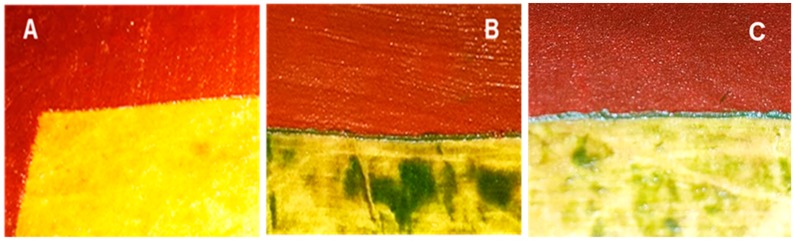
The *H. pluvialis* biofilm at initial dry biomass density of 2.5 g m^–2^ (**A**), 7.5 g m^–2^ (**B**) and 10 g m^–2^ (**C**) after 6 days of cultivation.

Here, we report that at a light intensity of 400–600 µmol photons m^−2^ s^−1^, initial biomass densities of 5–10 g dry weight m^−2^ yielded astaxanthin productivities of 160–180 mg m^−2^ d^−1^. Such biofilms revealed both an upper red and a lower green layer of cells after six days of growth (Figure 10B and 10C). At low initial biomass densities (2.5 g m^−2^) the biofilm consisted exclusively of red cells (Figure 10A), and astaxanthin productivities were significantly lower than at higher inoculum densities. Under the conditions chosen, 5–7.5 g m^−2^ appears to be an optimal initial biomass density for astaxanthin production in an angled TL-PSBR. The astaxanthin content in the dry biomass of this study (2–3 %) is in the same range as in many two-phase suspended cultivation systems but lower than the maximally reported contents in such systems [9,20,27,28]. This could be due to several factors including strain type, lack of high-energy (blue) photons in the artificial light source used, mixture of red and green cells, etc. 

### 4.2. Influence of Algal Storage Time

In the bench-scale vertical TL-PSBR setup of Kiperstok [18], if all 12 TL modules are operated at the same time, the total area of biofilms is about 12 × 24 × 2,5434 = 732.50 cm^2^ (each module has 24 plates, each plate has a diameter of 18 mm) so the amount of algae biomass to reach a density of 5 g m^−2^ will be only 0.366 g. This amount of biomass was easily collected by centrifuging the algal suspension for inoculation of the TL-PSBR. However, upon upscaling, the initial algae biomass needs to be large (about 30 g if two large-scale angled TL-PSBRs are simultaneously operated with a total inoculation area of 4 m^2^ to reach a density of 7.5 g m^−2^) and storage of algal biomass before inoculation becomes an issue. Therefore, the effect of algal storage time after centrifugation on subsequent growth and astaxanthin accumulation of *H. pluvialis* in TL-PSBRs was investigated in this study.

The most interesting result was obtained when the initial biomass was stored for 24 h at 4 °C before inoculation of the TL-PSBR. This yielded higher biomass and astaxanthin productivities compared to initial biomass that had not been stored. It is well known that upon exposure to low temperature (4 °C) *Haematococcus* predominantly exists in the non-motile palmella stage (e.g., as reported previously for an arctic strain by Klochkova et al. [29]). Non-flagellate green palmella stages of *Haematococcus* have a stronger tolerance to photooxidative stress [30] and could be more resistant to cell immobilization and/or may transform more successfully into akinetes. Longer storage times (>24 h) of the initial biomass, however, led to decreased biomass and astaxanthin productivities on TL-PSBRs presumably because of increased rates of cell death in the highly concentrated suspension.

### 4.3. Influence of Light Conditions and CO_2_ Supplement

Light intensities > 300 µmol photons m^−2^ s^−1^ increased dry biomass and astaxanthin accumulation of autotrophic *H. pluvialis* in TL-PSBRs in the presence of 5% (v/v) of CO_2_ added to the gas phase [23]. The results obtained in this study using different light intensities with the angled TL-PSBR generally corroborated the results of Kiperstok [18]. The somewhat lower biomass productivities (6-8 g biomass m^−2^ s^−1^ compared to 11-12 g biomass m^−2^ s^−1^ in Kiperstok et al. [23] under comparable conditions (500 µmol photons m^−2^ s^−1^ and 6 days of growth)) obtained in this study could be related to one or several of the following factors: the application of CO_2_ to the culture medium instead of the gas phase, slower medium flow in the angled PSBR compared to a vertical setup, the different substrate and source materials, the frequency of culture medium exchange, different methods of inoculation of the biomass to the TL-PSBR. Similarly, the astaxanthin productivities in the angled TL-PSBR were slightly lower (0.15–0.2 g m^−2^ d^−1^ compared to 0.2–0.3 g m^−2^ d^−1^) than those obtained by Kiperstok et al. in a vertical system [23]. However, astaxanthin productivity was higher than in most previous studies both in suspended and immobilized systems [7,9,19,20,27,28].

Under a light intensity range of 300–1000 µ mol photons m^−2^ s^−1^, the dry biomass productivity and astaxanthin accumulation were not significantly different between intensity points after 10 days of cultivation. This contrasts with the results of Kiperstok et al. [23], who showed that at 1000 µmol photons m^−2^ s^−1^, the total biomass content was ~130 g m^−2^ (compared to ~70 g m^−2^ in this study). This could be related to the higher CO_2_ concentrations used in Kiperstok et al. [23] and the different mode of CO_2_ application (in the gas phase vs. in the culture medium; see also discussion by Li et al. [31]). Further efforts should be made to optimize CO_2_ application to the algal biofilm. It should also be systematically investigated whether exchange of the culture medium with a nutrient (N and P)-depleted culture medium for the final 3 days of the 10 day cultivation period presents an advantage with respect to astaxanthin productivity compared to a longer (e.g., 16 day) cultivation period under nutrient-replete conditions (as in Kiperstok et al. [23]).

## 5. Conclusions

In conclusion, this study has shown that *H. pluvialis* can be grown successfully in a horizontal/low-angled TL-PSBR with acceptable biomass and astaxanthin productivities. Storage time and thus the physiological status of the initial biomass (inoculum) appears to have a significant effect on later growth and astaxanthin productivity of *H. pluvialis* in the TL-PSBR. A larger-scale angled TL-PSBR was successfully tested for pilot-scale applications.

## References

[1] Rao A.R., Sindhuja H.N., Dharmesh S.M., Sankar K.U., Sarada R., Ravishankar G.A. (2013). Effective inhibition of skin cancer, tyrosinase, and antioxidative properties by astaxanthin and astaxanthin esters from the green alga Haematococcus pluvialis. J. Agric. Food Chem..

[2] Gross G.J., Hazen S.L., Lockwood S.F. (2006). Seven day oral supplementation with Cardax (disodium disuccinate astaxanthin) provides significant cardioprotection and reduces oxidative stress in rats. Mol. Cell Biochem..

[3] Ambati R.R., Phang S.M., Ravi S., Aswathanarayana R.G. (2014). Astaxanthin: Sources, extraction, stability, biological activities and its commercial applications—A review. Mar. Drugs.

[4] Dong L.Y., Jin J., Lu G., Kang X.L. (2013). Astaxanthin attenuates the apoptosis of retinal ganglion cells in db/db mice by inhibition of oxidative stress. Mar. Drugs.

[5] Kang C.D., An J.Y., Park T.H., Sim S.J. (2006). Astaxanthin biosynthesis from simultaneous N and P uptake by the green alga *Haematococcus pluvialis* in primary-treated wastewater. Biochem. Eng. J..

[6] Lorenz R.T., Cysewski G.R. (2000). Commercial potential for *Haematococcus* microalgae as a natural source of astaxanthin. Trends Biotechnol..

[7] Zhang W., Wang J., Wang J., Liu T. (2014). Attached cultivation of *Haematococcus pluvialis* for astaxanthin production. Bioresour. Technol..

[8] Olaizola M., Huntley M., Fingerman M., Nagabhushanam R. (2003). Recent advances in commercial production of astaxanthin from microalgae. Biomaterials and Bioprocessing.

[9] Aflalo C., Meshulam Y., Zarka A., Boussiba S. (2007). On the relative efficiency of two- vs. one-stage production of astaxanthin by the green alga Haematococcus pluvialis. Biotechnol. Bioeng..

[10] Suh I.S., Joo H.N., Lee C.G. (2006). A novel double-layered photobioreactor for simultaneous *Haematococcus pluvialis* cell growth and astaxanthin accumulation. J. Biotechnol..

[11] Acién F.G., Molina E., Reis A., Torzillo G., Zittelli G.C., Sepúlveda C., Masojídek J., Gonzalez-Fernandez C., Muñoz R. (2017). 1—Photobioreactors for the production of microalgae. Microalgae-Based Biofuels and Bioproducts.

[12] Brennan L., Owende P. (2010). Biofuels from microalgae—A review of technologies for production, processing, and extractions of biofuels and co-products. Renew. Sustain. Energy Rev..

[13] Giuseppe O., Piero S., Antonio M. (2014). Advances in photobioreactors for intensive microalgal production: Configurations, operating strategies and applications. J. Chem. Technol. Biotechnol..

[14] Gross M., Jarboe D., Wen Z. (2015). Biofilm-based algal cultivation systems. Appl. Microbiol. Biotechnol..

[15] Naumann T., Çebi Z., Podola B., Melkonian M. (2013). Growing microalgae as aquaculture feeds on twin-layers: A novel solid-state photobioreactor. J. Appl. Phycol..

[16] Podola B., Li T., Melkonian M. (2017). Porous substrate bioreactors: A paradigm shift in microalgal biotechnology?. Trends Biotechnol..

[17] Nowack E.C.M., Podola B., Melkonian M. (2005). The 96-well twin-tayer system: A novel approach in the cultivation of microalgae. Protist.

[18] Kiperstok A.C. (2016). Optimizing immobilized cultivation of *H**aematococcus pluvialis* for astaxanthin production. Ph.D. Thesis.

[19] Wan M., Hou D., Li Y., Fan J., Huang J., Liang S., Wang W., Pan R., Wang J., Li S. (2014). The effective photoinduction of *Haematococcus pluvialis* for accumulating astaxanthin with attached cultivation. Bioresour. Technol..

[20] Wan M., Zhang J., Hou D., Fan J., Li Y., Huang J., Wang J. (2014). The effect of temperature on cell growth and astaxanthin accumulation of *Haematococcus pluvialis* during a light-dark cyclic cultivation. Bioresour. Technol..

[21] Yin S., Wang J., Chen L., Liu T. (2015). The water footprint of biofilm cultivation of *Haematococcus pluvialis* is greatly decreased by using sealed narrow chambers combined with slow aeration rate. Biotechnol. Lett..

[22] Shi J., Podola B., Melkonian M. (2007). Removal of nitrogen and phosphorus from wastewater using microalgae immobilized on twin layers: An experimental study. J. Appl. Phycol..

[23] Kiperstok A.C., Sebestyén P., Podola B., Melkonian M. (2017). Biofilm cultivation of *Haematococcus pluvialis* enables a highly productive one-phase process for astaxanthin production using high light intensities. Algal Res..

[24] Tran H.D., Do T.T., Le T.L., Tran-Nguyen M.L., Pham C.H., Melkonian M. (2019). Cultivation of *Haematococcus pluvialis* for astaxanthin production on angled bench-scale and large-scale biofilm-based photobioreactors. Vietnam J. Sci. Technol. Eng..

[25] Li Y., Miao F., Geng Y., Lu D., Zhang C., Zeng M. (2012). Accurate quantification of astaxanthin from *Haematococcus* crude extract spectrophotometrically. Chin. J. Oceanol. Limnol..

[26] Wang B., Zhang Z., Hu Q., Sommerfeld M., Lu Y., Han D. (2014). Cellular capacities for high-light acclimation and changing lipid profiles across life cycle stages of the green alga *Haematococcus pluvialis*. PLoS ONE.

[27] Torzillo G., Goksan T., Faraloni C., Kopecky J., Masojídek J. (2003). Interplay between photochemical activities and pigment composition in an outdoor culture of *Haematococcus pluvialis* during the shift from the green to red stage. J. Appl. Phycol..

[28] Zhang B.Y., Geng Y.H., Li Z.K., Hu H.J., Li Y.G. (2009). Production of astaxanthin from Haematococcus in open pond by two-stage growth one-step process. Aquaculture.

[29] Klochkova T., Seok Kwak M., Han J.W., Motomura T., Nagasato C., Kim G.H. (2013). Cold-tolerant strain of *Haematococcus pluvialis* (Haematococcaceae, Chlorophyta) from Blomstrandhalvøya (Svalbard). Algae.

[30] Li F., Cai M., Lin M., Huang X., Wang J., Ke H., Zheng X., Chen D., Wang C., Wu S. (2019). Differences between Motile and Nonmotile Cells of *Haematococcus pluvialis* in the Production of Astaxanthin at Different Light Intensities. Mar. Drugs.

[31] Li T., Strous M., Melkonian M. (2017). Biofilm-based photobioreactors: Their design and improving productivity through efficient supply of dissolved inorganic carbon. FEMS Microbiol. Lett..

